# Hip joint and muscle loading for persons with bilateral transfemoral/through-knee amputations: biomechanical differences between full-length articulated and foreshortened non-articulated prostheses

**DOI:** 10.1186/s12984-023-01296-4

**Published:** 2023-12-19

**Authors:** Diana Toderita, Clement D. Favier, David P. Henson, Vasiliki Vardakastani, Kate Sherman, Alexander N. Bennett, Anthony M. J. Bull

**Affiliations:** 1 Department of Bioengineering, Imperial College London, London, UK; 2 Dorset Orthopaedic GB, Manchester, UK; 3 Defence Medical Rehabilitation Centre, Loughborough, UK

**Keywords:** Lower limb loss, Transfemoral, Prosthesis, Joint degeneration, Muscular endurance

## Abstract

**Background:**

Currently, there is little available in-depth analysis of the biomechanical effect of different prostheses on the musculoskeletal system function and residual limb internal loading for persons with bilateral transfemoral/through-knee amputations (BTF). Commercially available prostheses for BTF include full-length articulated prostheses (microprocessor-controlled prosthetic knees with dynamic response prosthetic feet) and foreshortened non-articulated stubby prostheses. This study aims to assess and compare the BTF musculoskeletal function and loading during gait with these two types of prostheses.

**Methods:**

Gait data were collected from four male traumatic military BTF and four able-bodied (AB) matched controls using a 10-camera motion capture system with two force plates. BTF completed level-ground walking trials with full-length articulated and foreshortened non-articulated stubby prostheses. Inverse kinematics, inverse dynamics and musculoskeletal modelling simulations were conducted.

**Results:**

Full-length articulated prostheses introduced larger stride length (by 0.5 m) and walking speed (by 0.3 m/s) than stubbies. BTF with articulated prostheses showed larger peak hip extension angles (by 10.1°), flexion moment (by 1.0 Nm/kg) and second peak hip contact force (by 3.8 bodyweight) than stubbies. There was no difference in the hip joint loading profile between BTF with stubbies and AB for one gait cycle. Full-length articulated prostheses introduced higher hip flexor muscle force impulse than stubbies.

**Conclusions:**

Compared to stubbies, BTF with full-length articulated prostheses can achieve similar activity levels to persons without limb loss, but this may introduce detrimental muscle and hip joint loading, which may lead to reduced muscular endurance and joint degeneration. This study provides beneficial guidance in making informed decisions for prosthesis choice.

## Background

The Afghanistan conflict presented a total of 265 major lower limb United Kingdom military amputations from 2003 to 2014 [1]. Whilst the injured personnel sustained different locations, levels and number of amputations, the most common type of amputation was bilateral and transfemoral [1]. The post-injury musculoskeletal function is disrupted as the loss of joints, muscle volume and physiological muscle attachments lead to muscular adaptations [2] and compensatory strategies required for successfully completing activities of daily living. For example, the loss of ankle plantar flexors’ function during double limb support and at the end of the stance phase of the gait cycle leads to an increased burden on the hip flexor muscles at this stage of the gait cycle [3, 4]. The newly adopted compensatory strategies may lead to elevated muscle and joint contact forces for persons with bilateral amputations (BTF) compared to persons without limb loss [5]. For persons with unilateral transfemoral amputations, previous literature presented increased hip joint moments on the amputated side [6], as well as increased low back moments [7], compared to persons without limb loss, indicating that amputation may influence the loading of the residual joints. High and repetitive loading of the joints has been associated with increased risk of developing osteoarthritis [8, 9, 10], which is commonly reported for people with lower limb loss [11, 12]. Additionally, elevated muscle activations may increase the functional demand of residual muscles, leading to muscular fatigue [13, 14] and limited ability to ambulate [15].

Return to physical activity after amputation is achieved using prosthetic devices and rehabilitation. Commercially available prostheses for BTF include the foreshortened non-articulated stubbies and full-length articulated prostheses: microprocessor-controlled knee units coupled with dynamic response feet. The complex microprocessor knees can reproduce the power absorption phases of gait in the knee [16] and replicate the eccentric function of the quadriceps at heel strike and early swing, and of the hamstrings at the end of the swing phase. Following bed rest and wheelchair use post amputation and any additional surgical interventions, stubbies are used for immediate gait training of BTF. Once successful in efficiently walking with stubbies, BTF proceed to walk with full-length articulated prostheses. However, even if able to walk with full-length articulated prostheses, there is anecdotal evidence that some BTF choose to use stubbies when they feel tired.

Previous studies evaluated the functional performance of military traumatic BTF and showed high functional levels with effective gait patterns [6, 17], but using 60% more oxygen than persons without limb loss to achieve the same outcomes [17]. However, these studies did not analyse the biomechanical effect of different prosthetic designs on the BTF musculoskeletal function and loading, as all participants were fitted with microprocessor knees and dynamic response feet. The biomechanics with stubby prostheses has been previously presented in case studies, which showed slower walking speeds [18, 19] and higher oxygen cost when used for long distances [20] compared to conventional full-length articulated prostheses. Previous studies focused on spatiotemporal, metabolic, kinematics and dynamic measures, excluding other relevant physiological aspects such as muscle and joint contact forces, that are related to pathology. This is the first study to thoroughly investigate biomechanical differences (functional abilities, joint kinematics, kinetics and contact forces) between full-length articulated and foreshortened non-articulated stubby prostheses for BTF. Joint contact forces can be used to assess joint health [21] and muscle force impulse (area under the force-time curve) can be used to assess endurance [22]. Musculoskeletal modelling is a widely used tool that can provide comprehensive descriptions of muscle and joint contact forces during movement [23–25]. The analysis of functional, as well as biomechanical loading measures, allows for the development of strategies to reduce the risk of musculoskeletal health-related complications, whilst also considering functional performance.

The aim of this study is to use biomechanical tools to understand the musculoskeletal function and loading of persons with BTF amputations who can walk with full-length articulated and foreshortened non-articulated stubby prostheses. The findings of this study will provide guidance in making informed decisions for adequate prosthesis choice to ensure optimal rehabilitation and long-term musculoskeletal health.

## Methods

This study received approval from the institutional ethics review board (Imperial College Research Ethics Committee, Reference 20IC6268). Four male traumatic BTF and four group matched able-bodied (AB) males with no known musculoskeletal or neurological condition took part in the study. Written informed consent was obtained from all participants, whose details are presented in Table 1. All BTF have undergone comprehensive rehabilitation at the Defence Medical Rehabilitation Centre UK, had been regularly using both full-length articulated prostheses and non-articulated stubbies, and had a K3 activity level or higher. BTF were group matched to AB persons based on adjusted mass for amputation (calculated according to literature [26]) and pre-injury height. There were no significant differences in age (p = .999), adjusted mass (p = .886) and pre-injury height (p = .686). This was checked with Mann-Whitney U tests with a 0.05 significance level.

**Table 1:** Study participant details

Participant^a^	Age (years)	Mass (kg)^b^	Height (cm)^c^	Stump length (cm)		Cause of amputation	Time since amputation (years)
				Left	Right		
BTF 1	36	89	187	55	44	IED	9
BTF 2	32	68	171	40	41	IED	9
BTF 3	34	84	180	36	47	IED	11
BTF 4	41	86	187	50	33	IED	11
Mean ± SD	36 ± 3	82 ± 8	181 ± 7	45 ± 8)	41 ± 5)	N/A	10 ± 1)
AB controls Mean ± SD	35 ± 3	82 ± 7	182 ± 2	N/A	N/A	N/A	N/A

IED, improvised explosive device

^a^All participants are male

^b^Adjusted mass for amputation

^c^Pre-injury height

The biomechanical model, described in more detail later, necessitated motion and force plate data to compute joint kinematics, kinetics, and contact forces. Motion data were collected at an acquisition rate of 120 Hz using a 10-camera VICON motion analysis system (VICON 2.10.3, Oxford Metrics Group, UK). The force plate data were collected with two force plates (Kistler Type 9286B, Kistler Instrumente AG, Winterthur, Switzerland) at an acquisition rate of 1000 Hz. Retro-reflective markers were placed on the lower limb anatomical landmarks detailed in Table 2.

**Table 2:** Marker placement locations

Marker name	Marker location
RASIS/LASIS	Right/Left anterior superior iliac spine
RPSIS/LPSIS	Right/Left posterior superior iliac spine
RFME/LFME	Right/Left medial femoral epicondyle Prosthetic knee equivalent: medial side of the knee centre of rotation Stubbies equivalent: medial side of the pylon, below the prosthetic socket
RFLE/LFLE	Right/Left lateral femoral epicondyle Prosthetic knee equivalent: lateral side of the knee centre of rotation Stubbies equivalent: lateral side of the pylon, below the prosthetic socket
RFAM/LFAM	Right/Left lateral malleoli Prosthetic equivalent: lateral side of the ankle centre of rotation
RTAM/LTAM	Right/Left medial malleoli Prosthetic equivalent: medial side of the ankle centre of rotation
RFM2/LFM2	Right/Left second metatarsal head Prosthetic equivalent: front edge of the foot above the second toe
RFM5/LFM5	Right/Left fifth metatarsal head Prosthetic equivalent: front edge of the foot above the fifth toe
RFCC/LFCC	Right/Left posterior calcaneus Prosthetic equivalent: heel at toe height
RTF/LTF	Right/Left midfoot superior Prosthetic equivalent: top of the foot, anterior to the pylon base
R/L T1/T2/T3	Right/Left thigh segment cluster
R/L C1/C2/C3	Right/Left shank segment cluster

Static calibration and level ground self-selected speed walking trials, with complete foot placement on the force plate, were collected for all study participants with both stubby prostheses and microprocessor-controlled prosthetic knees coupled with dynamic response prosthetic feet. The able-body control dataset is a subgroup of a previous dataset [6]. VICON Nexus (VICON 2.10.3, Oxford Metrics Group, UK) was used to identify the gait cycle events (heel strike, toe off, consecutive ipsilateral heel strike) using a 30 N force threshold, label the reflective markers and fill the marker trajectory gaps using the rigid body gap filling algorithm. The marker trajectories and ground reaction forces were filtered in MATLAB (The Mathworks Inc., Natwick, MA, USA) using a zero phase-lag, fourth order Butterworth filter with 6 Hz cut-off frequency [25, 27, 28] prior to the musculoskeletal model input.

The biomechanical variables for the left and right limbs for each participant were not considered independent as the two lower limb models share the same pelvic segment and associated pelvic bone and muscle characteristics. Therefore, to avoid bias of the results, all biomechanical parameters were presented for the right limb only for all study participants.

Freebody v2.1 was used to perform inverse kinematics, inverse dynamics and musculoskeletal modelling simulations adapted for able-body [24, 29] and transfemoral amputation use, presented in previous literature [5, 30]. Briefly, Freebody v2.1 is comprised of four rigid segments (foot, shank, thigh, pelvis) and three joints (ankle, knee, hip). The ankle and knee joints possess three rotational and three translational degrees of freedom (DOFs), and in this use of the model, the hip was constrained to three rotational DOFs. In the transfemoral model, the stump, prosthetic liner, socket, and connector were combined to model the thigh segment and reduce the computational complexity. To determine the prosthetic device’s influence on the overall joint moment, inverse dynamics was employed. The torque values were reported at the midpoint between the medial and lateral epicondyles for the prosthetic knee and between the medial and lateral malleoli for the prosthetic foot. The muscles spanning the missing joint were either removed or repositioned to accommodate their post-amputation attachment points. Two musculoskeletal models were created for each BTF participant: one model with stubbies and one with full-length prostheses, leading to a total of eight transfemoral musculoskeletal models. Six models were subject-specific, where the joint and muscle parameters were obtained from a previous MRI scans analysis [30], thus ensuring the accuracy of the muscle and hip joint contact force predictions. The anatomical geometry for the right limb of the remaining transfemoral model was obtained through linear scaling to an anatomical dataset chosen based on similar pelvis width, estimated intact body mass index (calculated using the intact mass [26]) and right stump length to pelvis width ratio to accurately predict muscle and hip joint contact forces [30]. Similarly, the closest datasets for the able-body models were chosen based on similar mass, gender and limb length to pelvis width ratio [31].

The one-step static load sharing optimisation algorithm presented in Eq. 1 [32] was used to compute the forces in each muscle element (${F}_{i}$) and the resultant contact force at the hip joint by minimizing the sum of cubed muscle activations (J). M represents the total number of muscle elements, where M = 92 for the transfemoral model and M = 163 for the able-body model. ${F}_{max,i}$ represents the maximum capacity force for each muscle element, and was defined as the multiplication of the muscle element physiological cross-sectional area and maximum muscle stress (31.39 N/cm^2^ [33]).

1 $$\text{min}\left(J\right)=\text{m}\text{i}\text{n}\left({\sum _{i=1}^{M}\left(\frac{{F}_{i}}{{F}_{max,i} }\right)}^{3}\right)$$

The analysis for each participant included 3 strides, where the computed whole-time data series were expressed as a gait cycle percentage from 0% at heel strike to 100% at the consecutive ipsilateral heel strike in intervals of 1% and averaged over three trials. Descriptive statistics only were used due to the sample size.

## Results

Table 3 presents the temporospatial characteristics for BTF with stubbies and full-length articulated prostheses and AB controls. BTF showed larger stride lengths by 0.5 m with full-length articulated prostheses than stubbies. Whilst the walking speed was faster by 0.3 m/s with full-length articulated prostheses, cadence was lower by 12.6 steps/min than stubbies. Except for the adopted step width, which was significantly higher for BTF with both prostheses than AB, BTF with full-length articulated prostheses showed similar temporospatial characteristics to AB.

**Table 3:** Temporal and spatial characteristics

Biomechanical parameter	BTF stubbies Mean ± SD	BTF full-length prostheses Mean ± SD	AB Mean ± SD
Step width (m)	0.23 ± 0.03	0.22 ± 0.03	0.09 ± 0.03
Stride length (m)	0.96 ± 0.13	1.46 ± 0.07	1.48 ± 0.05
Cadence (steps/min)	114.74 ± 3.90	102.17 ± 2.48	103.78 ± 2.04
Walking speed (m/s)	0.93 ± 0.10	1.22 ± 0.06	1.28 ± 0.07
Stance time (% cycle)	61.56 ± 1.19	64.35 ± 1.42	65.32 ± 1.30

**Fig. 1:**
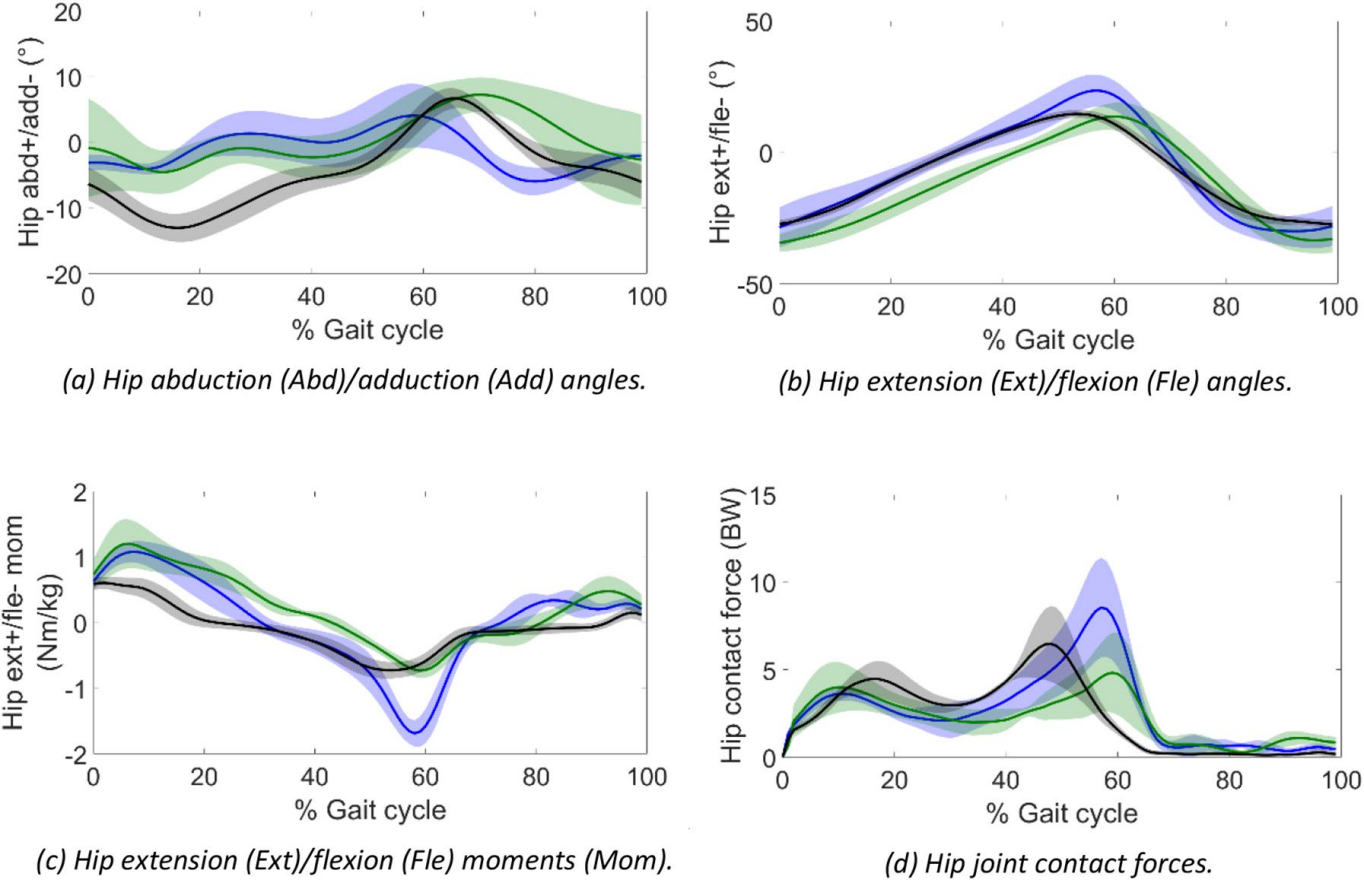
Hip angles, moments, and contact forces for BTF using two different prosthetic designs and AB. BTF stubbies – green. BTF full-length articulated prostheses – blue. AB – black. Shaded regions represent standard deviation

Figure 1 and Table 4 present the hip kinematics and kinetics over the course of the gait cycle. BTF showed higher hip abduction angles than AB in stance and similar between prostheses at this stage of the gait cycle. However, the swing phase of the gait cycle presented lower hip abduction angles with full-length articulated prostheses than stubbies. The hip extension angles at the end of stance were higher with full-length prostheses by 10.1° than stubbies and by 9.2° than AB, and these led to larger peak hip flexion moments by 1.0 Nm/kg with full-length articulated prostheses than stubbies and AB. The second peak of the hip joint contact forces (HJCF) was larger with full-length articulated prostheses than stubbies by 3.8 BW. Although BTF with stubbies and AB presented similar HJCF profiles during one gait cycle, the HJCF impulse normalised to walked distance was higher for BTF with stubbies by 61.6 BW.s/m than AB, but not different than BTF with full-length articulated prostheses.

**Table 4:** Peak hip joint angles, moments, contact forces and impulse for BTF using two different prosthetic designs

Biomechanical parameter	BTF stubbies Mean ± SD	BTF full-length articulated prostheses Mean ± SD	AB Mean ± SD
Peak hip extension (°)	13.81 ± 5.23	23.94 ± 5.92	14.78 ± 1.45
Peak hip flexion moment (Nm/kg)	0.75 ± 0.12	1.78 ± 0.27	0.77 ± 0.11
First peak hip contact force (BW)	4.06 ± 1.45	3.67 ± 0.37	4.34 ± 0.61
Second peak hip contact force (BW)	5.25 ± 2.15	9.07 ± 2.87	5.43 ± 0.52
Hip contact force impulse (BW.s)	263.68 ± 58.61	350.41 ± 61.43	309.71 ± 26.57
Normalised hip contact force impulse (BW.s/m)	272.63 ± 30.52	239.36 ± 34.70	211.02 ± 18.10

Table 5 presents the calculated hip flexor muscle force impulse and impulse normalised to walked distance for BTF with full-length articulated prostheses and stubbies. Compared to stubbies, walking with full-length articulated prostheses showed larger impulse for iliacus (by 16.2 BW.s), psoas (by 20.7 BW.s), rectus femoris (by 4.9 BW.s) and sartorius (by 1.7 BW.s). No significant muscle force impulse differences were identified between prostheses after normalisation to distance walked.

**Table 5:** Hip flexor muscle force impulse and impulse normalised to walked distance for BTF using two different prosthetic designs

Muscle	BTF stubbies Mean ± SD		BTF full-length articulated prostheses Mean ± SD	
	Impulse (BW.s)	Normalised impulse (BW.s/m)	Impulse (BW.s)	Normalised impulse (BW.s/m)
Iliacus	19.51 ± 11.14	19.80 ± 9.80	35.71 ± 13.13	24.23 ± 7.92
Psoas	19.60 ± 11.51	20.02 ± 11.23	40.29 ± 15.15	27.28 ± 9.18
Rectus femoris	4.49 ± 2.67	4.48 ± 2.27	9.34 ± 3.14	6.34 ± 1.84
Sartorius	1.54 ± 0.87	1.55 ± 0.81	3.23 ± 0.27	2.22 ± 0.15

## Discussion

This is the first study to comprehensively investigate differences in function and loading of the BTF musculoskeletal system during level-ground walking with two different types of prostheses: foreshortened non-articulated stubbies and full-length articulated prostheses (microprocessor-controlled prosthetic knees coupled with dynamic response feet).

BTF walked with larger step widths than AB, in accordance with previous literature on BTF gait with full-length articulated prostheses [17]. Increasing the step width reduces the hip adduction moment at full hip extension to better mechanically recruit the abductor muscles, and increases stability and power production capabilities [34]. Metabolic cost has been strongly correlated to the adopted step width [35], which might explain how the use of prosthetic devices may increase metabolic energy expenditure and reduce efficiency if used for walking long distances and periods of time, as presented in previous literature data [17].

Albeit severely injured, BTF demonstrated with full-length articulated prostheses stride lengths, cadence and walking speeds similar to those of AB controls, as previously seen in the literature [17]. However, BTF showed lower functional abilities (as determined by walking speed) with stubbies than full-length articulated prostheses. Interestingly, participants showed higher cadence with stubbies than full-length articulated prostheses and AB. This may indicate an attempt to maintain similar walking speeds to those achieved with prosthetic knees by increasing cadence [19], which was not sufficient given the lower stride lengths with stubbies. Unlike stubbies, BTF with full-length articulated prostheses needed to adopt larger stride lengths to be able to swing the full-length prosthesis back from a larger hip extension position, which ultimately created higher hip flexion moments, burdening the hip flexor muscles, in accordance with previous literature that demonstrated high hip flexors activations during gait compared to persons without limb loss [3]. The elevated hip joint moments have also been reported for the amputated limb of persons with unilateral transfemoral amputations with prosthetic sockets [6, 36], which indicates that the observed kinetics might be a consequence of the level of amputation, amongst other factors such as preferred movement strategies, prosthesis characteristics and socket fit. As an alternative method to fix the prosthesis to the residual limb, osseointegration has been shown to reduce the amputated side hip joint moments and contact forces for persons with unilateral transfemoral amputations [36]. Future research could investigate the biomechanical effect of osseointegration on the BTF hip joint loading, and how this differs between stubbies and full-length articulated prostheses.

Although BTF with full-length prostheses were able to achieve similar functional levels in gait to persons without limb loss, they experienced significantly larger hip joint contact forces (HJCF). Conversely, the hip loading profile of BTF with stubbies was comparable to that of AB controls. BTF with full-length articulated prostheses had a second HJCF peak that was nearly double than that observed with stubbies. However, walking speed has a direct effect on the second HJCF peak [37], and BTF with stubbies walked at slower speeds than full-length articulated prostheses. The faster walking speed of BTF with full-length articulated prostheses, as well as the higher centre of gravity from the ground, may have required higher muscle co-contraction, which in turn increased the hip joint loading magnitude. However, muscle co-contraction is not represented in static-optimization based musculoskeletal models, and the effect of this limitation could be investigated.

Osteoarthritis is a complex, multifactorial disease, with biomechanical and biological factors playing a significant role in osteoarthritis pathogenesis [38, 39]. From a mechanical perspective, the risk of osteoarthritis for persons without limb loss and persons with unilateral transfemoral and transtibial amputations has been linked to high and repetitive joint loading [21, 25, 40, 41]. A previous longitudinal study used logistic regression analysis to show that the risk of knee osteoarthritis progression for persons without limb loss increases 6.46 times with a 1% increase in knee adduction moment [8]. Additionally, in a cross-sectional analysis, the medial knee contact forces were significantly higher for persons without limb loss with severe medial knee osteoarthritis compared to other severity grades [10]. To our knowledge, the relation between mechanical loading and the risk of hip osteoarthritis has not been investigated for people with and without amputations, and future work could study this. Based on the previously developed causal links between knee loading and osteoarthritis development, it is assumed that higher and more repetitive loading compared to AB control data may increase the risk of hip osteoarthritis. In this study, although stubbies reduced the magnitude of the maximum force at the hip during one gait cycle, the hip joint force impulse normalised to walked distance was higher than AB. This result may indicate that use of stubby prostheses for long distance and times might not be beneficial for bone and joint health. Stubby prostheses may introduce similar hip loading profiles to those of AB only if they are used for walking short distances, training and use around the house. The higher hip joint contact forces and impulse during one gait cycle with full-length prostheses compared to stubbies and AB suggest that for short term use, high-functioning BTF may be more susceptible to hip joint degeneration onset with full-length articulated prostheses than foreshortened non-articulated stubbies. Therefore, there is a balance between the appropriateness of each prosthesis based on the type of performed activity. However, the small cohort limits the use of these results to a wider BTF population, and a greater sample size is required for adequate generalization. Variations in height, body mass and gender could be considered in future studies. In the context of this study, the number of study participants was as large as could be achieved.

In contrast to able-body baseline, muscular activation changes after amputation due to anatomical factors, prosthetic requirements and adopted biomechanics. The larger hip flexion moments with full-length articulated prostheses contributed to higher hip flexor muscular effort compared to stubbies, as demonstrated by the larger muscle force impulse, used as an indication of muscular endurance [22]. The observed hip flexor muscle impulse values per one stride suggest that use of full-length articulated prostheses may lead to reduced muscular endurance in comparison to stubbies. However, given the shorter stride lengths, more strides are required with stubbies to reach the same distance as full-length prostheses, and so, the muscle force impulse normalised to walked distance is similar between prostheses. This again indicates that stubbies may be adequate for short-term over short distances use only.

There are limitations to this study. The sample size limitations meant that no statistical analysis was conducted, thus limiting the generalisability of the descriptive results. The model assumed that the external load is axially transmitted through the distal end of the residual limb. Whilst this is representative of participants with end-bearing quadrilateral sockets, ischial containment sockets distribute the weight more proximally to the ischium. Three participants used quadrilateral end-bearing sockets and one participant used ischial containment sockets. Additionally, the average time between the MRI scans and gait data collection was 2.7 years, and potential changes in muscle physiology (such as muscle hypertrophy, atrophy, and tissue fattening) might have affected the calculated muscle volume and ultimately, the muscle force predictions from the musculoskeletal model. However, as these potential anatomical changes could not have affected the bony landmarks, muscle origin and insertion coordinates, which are key in the musculoskeletal model, the effect of this limitation might not be significant. Future studies could investigate this. Other inherent modelling limitations are presented in previous work [30].

## Conclusion

This study highlights differences in musculoskeletal function and loading in BTF with foreshortened non-articulated stubbies and full-length articulated prostheses (microprocessor-controlled knees coupled with dynamic response feet) during level-ground walking in a group of participants who underwent the same rehabilitation care at the Defence Medical Rehabilitation Centre UK. Based on this study, it can be concluded that prosthesis choice should be based on activity levels, goals, musculoskeletal health factors and cosmesis preference. The balance between these varies per individual.

## Data Availability

The datasets used and analysed during the current study are available from the corresponding author on reasonable request.

## Abbreviations

BTF: bilateral above/through-knee amputation
AB: able-bodied
UK: United Kingdom
DOF: degree of freedom
BW: bodyweight
Abd: abduction
Add: adduction
Ext: extension
Fle: flexion
Mom: moment
SD: standard deviation
